# The Way to a Man's Heart Is through His Stomach: What about Horses?

**DOI:** 10.1371/journal.pone.0015446

**Published:** 2010-11-15

**Authors:** Carol Sankey, Séverine Henry, Aleksandra Górecka-Bruzda, Marie-Annick Richard-Yris, Martine Hausberger

**Affiliations:** 1 Ethologie Animale et Humaine, Université Rennes1/UMR-CNRS6552, Station Biologique, Paimpont, France; 2 Polish Academy of Sciences, Institute of Genetics and Animal Breeding, Jastrzębiec, Wólka-Kosowska, Poland; Université Pierre et Marie Curie, France

## Abstract

**Background:**

How do we bond to one another? While in some species, like humans, physical contact plays a role in the process of attachment, it has been suggested that tactile contact's value may greatly differ according to the species considered. Nevertheless, grooming is often considered as a pleasurable experience for domestic animals, even though scientific data is lacking. On another hand, food seems to be involved in the creation of most relationships in a variety of species.

**Methodology/Principal Findings:**

In this study, we used the horse training context to test the effects of food *versus* grooming during repeated human-horse interactions. The results reveal that food certainly holds a key role in the attachment process, while tactile contact was here clearly insufficient for bonding to occur.

**Conclusion/Significance:**

This study raises important questions on the way tactile contact is perceived, and shows that large inter-species differences are to be expected.

## Introduction

How do we bond to each other? What is it that leads to the process of attachment? In psychology, bonding is defined as the process of development of a close, interpersonal relationship [1]. Bonding typically refers to the process of attachment that develops between romantic partners, close friends or parents and children, but has also been used for human-animal relationships [2]. There is evidence that oxytocin and vasopressin hormones are involved in the bonding process and in other forms of prosocial and reproductive behaviour [3]. Of all bonds, the maternal (mother-infant) bond is one of the strongest, in which suckling (*i.e.* breastfeeding in humans) has been reported to have a fostering role [4], [5]. Animal research has shown that the endocrine response to suckling (oxytocin release) plays an essential role in maternal bonding by promoting maternal care-giving behavior [6]. Thus one of the strongest bonds in the animal kingdom is, at least partly, a feeding bond. Interestingly, the detachment in the feeding bond goes together with a detachment in the affectionate bond [7]. More generally, food sharing has been described as a reciprocal act of physical affiliation [8] and an essential component for the development of pair bond [9]. Food calls are another example of how food holds a prime position in the formation and maintenance of close relationships [10]. Don't we also say that little gifts keep friendship warm? Is there a better little gift than a box of sweets or chocolates to make a lover's heart melt or fill a grandmother with joy?

However, inter-individual bonding is often described in terms of social interactions and physical contacts. For this may well reflect real bonding in some species (*e.g.* in humans: [11], in cats: [12]), the value of tactile contact may differ according to the species considered. The same questioning arises when considering human-animal relationships. Even though it is clear that the taming process can be achieved by positive association conditioning: humans being the providers of food and water for domestic animals, they become secondarily associated with those positive stimuli [13], many still use diverse forms of tactile contact (e.g. stroking, grooming) to initiate bonding. While for the domestic dog, the human presence itself may be rewarding [13], more precocial species seem to have a less positive perception of human contact [14]. Nevertheless, grooming is often believed to be and used as a primary reinforcement, partly because it has been shown to induce a decrease in the groomee's heart rate [15], [16]. Here, we investigated whether grooming could be used to promote bonding and facilitate learning, by comparing it to a food-reward that has proven efficient for both [17], [18].

## Materials and Methods

Experiments complied with the current French and Polish laws related to animal experimentation and were in accordance to the European directive 86/609/CEE. This experiment only included behavioural observations, routine training and non-invasive contacts with the horses (giving carrots or scratching the withers) which did not require the approval of an ethics committee. Animal husbandry and care were under management of the staff of the research station in Popielno.

Study subjects were 20 Konik horses, a primitive breed originating directly from the wild Tarpan horse [19]. Subjects were reared under either conventional domestic conditions (N = 12) or with their respective families in semi-natural conditions in a 1600 ha forest reserve (N = 8). Forest-reared youngstock was caught and put together with their stabled peers at about 10 months. All weanlings were then kept together in multi-age groups, where they were able to express their natural behavioural repertoire in which grooming the withers is considered to play a socio-positive role [20]. Horses were aged one to two years old at the time of the experiment and housed in loose stables. No additional contact with humans took place, except for daily tethering for feeding. Horses were randomly allocated to one of two training groups:

- food-reward group (FR: N = 10): the experimenter hand-gave a small piece of carrot to the horse when it responded correctly to her command.
- grooming-reward group (GR: N = 10): the experimenter vigorously scratched the horse's withers three times when it responded correctly to her command.

Subjects underwent a training program to learn to remain immobile in response to a vocal command: “reste!”, for an increasing duration (5 to 60 seconds). Training was performed 5 min per day for 6 days and took place in the horses' home stable, where they were tethered facing the walls and given hay *ad libitum*. For training, the experimenter led the focal horse to the center of the stable. In order to distract the awaiting horses' attention from the vocal command, a white noise was broadcast *via* two radios placed each side of the stable, facing the tethered horses. After completion of daily training, they were set free in an adjacent outdoor paddock. The data collected during training included the maximum time step validated (*i.e.* 3 consecutive successes) each day, as well as the maximum time for which the horse remained immobile.

In addition, we performed a “motionless human test”, commonly used in the literature to assess human-animal relationships [21], before and after training. During 5 min, horses were free to interact with the person (female) standing in the center of the stable. Data collected included latency to approach her and total time spent at a distance of 0.5 m or less.

Non parametric statistics were used: Mann-Whitney *U*-tests (MW) to compare groups; Friedman (F) and Wilcoxon (W) tests to evaluate each group's progression. These analyses were conducted using Statistica© 7.1 software (accepted p level at 0.05).

## Results

Clear differences occurred both in learning performance and in the relationship to humans according to the type of “reinforcement” used. While on the last day of training almost all horses trained with the food reward had successfully reached the last step and managed to maintain immobility for 1 min, only 4 of the GR group did (*N*_FR_ = 9/10, *N*_GR_ = 4/10, Friedman test: *P* = 0.03; mean step reached on day6: _FR_±SE = 40±5.7 s, 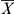
_GR_±SE = 18±6.1, MW: *U* = 20, *P* = 0.02).

**Figure 1:**
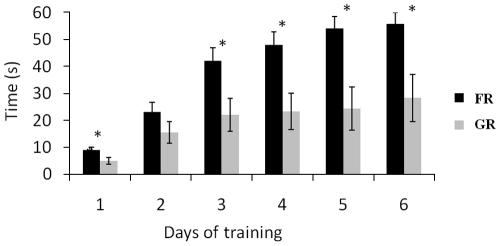
Maximum duration of immobility on order for food-rewarded and grooming-rewarded horses. Friedman tests: *P*<0.001, Mann-Whitney *U*-test, *P<0.05.

FR horses progressed rapidly, especially during the first three days of training: the maximum duration of immobility greatly increased from day1 to day2 (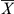
_day1_±SE = 9±1, 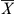
_day2_±SE = 23±3.7, W: *t* = 0, *P* = 0.02) and from day2 to day3 (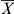
_day2_±SE = 23±3.7, 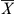
_day3_±SE = 42±4.5, W: *t* = 0, *P* = 0.03) and reached a mean duration of 55.7±4.3 s on the last day (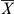
_day3_±SE = 42±4.5, 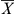
_day6_±SE = 57±3.6 s, n = 10, W: *t* = 0, *P* = 0.04). On the contrary, GR horses' progression was limited to the first two days of training (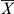
_day1_±SE = 5±1.3, 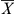
_day2_±SE = 15.5±4, W: *t* = 0, *P* = 0.02) after what they stagnated (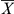
_day2_±SE = 15.5±4, 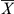
_day6_±SE = 31.5±8.9, W: *P* = 0.08; **fig. 1**). FR horses almost always managed to maintain immobility longer than GR horses in response to the order (**fig. 1**).

**Figure 2:**
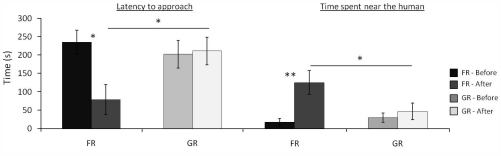
Latency to approach the experimenter and time spent near her (distance <0.5 m) in the “motionless person test” by food-rewarded and grooming-rewarded horses, before and after training. Mann-Whitney *U*-test & Wilcoxon test *P<0.05, **P<0.01.

More interesting is that while FR training had a positive impact on the relationship (shorter latency to approach the human: 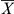
_before_±SE = 235.6±32.7, 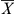
_after_±SE = 78.8±37.7 s, n = 10, W: *t* = 3, *P* = 0.02, more time spent near her, 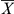
_before_±SE = 16.8±9.4, 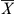
_after_±SE = 117.7±30.5 s, W: n = 10, *t* = 1, *P* = 0.01, after than before training), the grooming procedure had none (latency to approach: 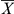
_before_±SE = 202.8±40.9, 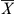
_after_±SE = 211.4±37.5 s, *P*>0.1, time spent near : 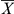
_before_±SE = 28±11.6, 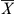
_after_±SE = 44±21.3 s, *P*>0.1), showing that in fact it is not a proper reinforcement. Secondly, while no difference in the horses' relation to humans was observed between groups before training, FR horses approached sooner than GR horses after training (MW, *U* = 15, *P* = 0.007), and also spent more time near (MW, *U* = 23.5, *P* = 0.04) (**fig. 2**).

## Discussion

Using food rewards had beneficial effects on horses' attachment to humans and facilitated learning, whereas the tactile contact was clearly not perceived sufficiently positively, neither for bonding to occur, nor for enhancing learning.

Food-rewarded animals learned the immobility task faster than grooming-rewarded animals. In fact, the performance of the latter on the sixth day of training was very close to that found in control horses trained to the same task in a previous study (28.7±3.7 s, [17]). Grooming the withers therefore does not appear to be an efficient reinforcement for horses. The positive value of human tactile contact sometimes described may in fact be acquired through association with other primary positive reinforcements such as food [22], [14], and should therefore be qualified of secondary reinforcement. Since Skinner's pioneering work [23], [24], food reinforcement has become one of the main incentives in conditioning procedures [25]. For example, scientists have obtained considerable success in training primates to cooperate during blood sample collection [26] or during various other handling or veterinary procedures [27]–[29]. Food rewards have been successfully used in a variety of conditioning paradigms and with a variety of species (*e.g.* in rats [30], dogs [31] or rhinoceros [32]).

Nevertheless, because we, humans, are sensitive to tactile stimulations [33], [11], we often assume that stroking or other forms of gentling animals have positive effects. This may indeed be the case for some species, but this study clearly demonstrates that interspecies differences are to be expected: the results suggest that human tactile contact, even when imitating intraspecific natural interactions, is not necessarily perceived positively and is surely not sufficient to create attachment [14]. In horses, physical contact is very restricted through occasional licking of the young by its dam and later mutual grooming, it only represents 2–3% of their time-budget [34] and is often restricted to specific body regions [15]. Studies have reported that grooming at the withers, whether performed by a conspecific or a human handler induced a decrease in horses' heart rate [15], [16]. However grooming was performed for a much longer duration (∼3 min) and a decrease in heart rate does not mean that it is perceived sufficiently positively to be considered as reinforcement and thus promote learning or bonding.

Moreover, the few and short food mediated interactions had a major positive effect on horse-human attachment: horses trained with a food reward approached sooner and were closer to humans. Proximities between horses are generally used to evaluate individual preferences and affinities [21]. Thus, food appears to be one of the keys in the bonding process [17], [18]. There is an idiomatic expression that says: “*the way to a man's heart is through his stomach*”. It seems that this may not only apply to humans, but could indeed be the case for many species, amongst which horses.
